# Clinical application of stem cell therapy in Parkinson's disease

**DOI:** 10.1186/1741-7015-10-1

**Published:** 2012-01-04

**Authors:** Marios Politis, Olle Lindvall

**Affiliations:** 1 Centre for Neuroscience, Department of Medicine, Imperial College London, Hammersmith Hospital, DuCane Road, London W12 0NN, UK; 2 Laboratory of Neurogenesis and Cell Therapy, Wallenberg Neuroscience Center and Lund Stem Cell Center, University Hospital, SE-221 84 Lund, Sweden

## Abstract

Cell replacement therapies in Parkinson's disease (PD) aim to provide long-lasting relief of patients' symptoms. Previous clinical trials using transplantation of human fetal ventral mesencephalic (hfVM) tissue in the striata of PD patients have provided proof-of-principle that such grafts can restore striatal dopaminergic (DA-ergic) function. The transplants survive, reinnervate the striatum, and generate adequate symptomatic relief in some patients for more than a decade following operation. However, the initial clinical trials lacked homogeneity of outcomes and were hindered by the development of troublesome graft-induced dyskinesias in a subgroup of patients. Although recent knowledge has provided insights for overcoming these obstacles, it is unlikely that transplantation of hfVM tissue will become routine treatment for PD owing to problems with tissue availability and standardization of the grafts. The main focus now is on producing DA-ergic neuroblasts for transplantation from stem cells (SCs). There is a range of emerging sources of SCs for generating a DA-ergic fate *in vitro*. However, the translation of these efforts *in vivo *currently lacks efficacy and sustainability. A successful, clinically competitive SC therapy in PD needs to produce long-lasting symptomatic relief without side effects while counteracting PD progression.

## Introduction

Parkinson's disease (PD) is a common neurodegenenerative disorder characterized by the classical motor symptoms of bradykinesia, rigidity and tremor. The pathological hallmark of PD is a gradual loss of nigostriatal dopamine (DA) neurons, but neuronal degeneration also occurs in non-DA-ergic systems [1]. Treatments aiming to relieve PD motor symptoms include the use of oral preparations of L-3,4-dihydroxyphenylalanine (L-DOPA) and DA receptor agonists and, in more advanced cases, the use of apomorphine, delivery of L-DOPA through continuous intestinal administration, and deep brain stimulation in subthalamic nucleus and globus pallidus *via *surgically implanted electrodes. These treatments have proved effective to a point, but they can generate adverse effects, such as L-DOPA-induced dyskinesias, and they do not counteract the progression of the disease.

**Figure 1:**
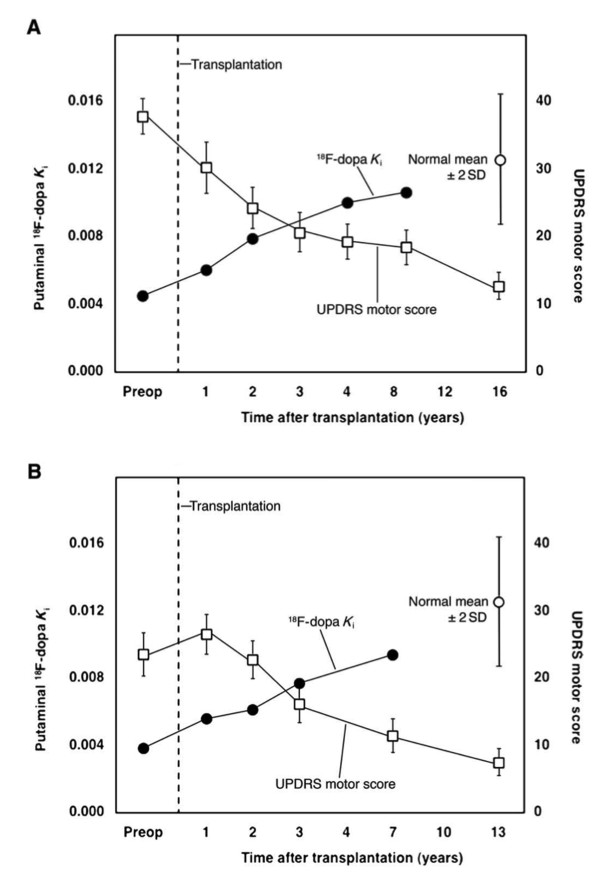
**Human fetal ventral mesencephalic tissue grafts provide long-lasting major relief of motor symptoms (reductions in UPDRS motor scores) and restore dopamine innervation (increases in 18F-DOPA PET uptake) in the grafted striatum in Patients 7 (A) and 15 (B) from the Lund series (modified figure from **[4]). Patient 7 and Patient 15 stopped receiving any form of dopaminergic medication four and five years following operation, respectively. PET = Positron emission tomography; UPDRS = Unified Parkinson's Disease Rating Scale.

**Figure 2:**
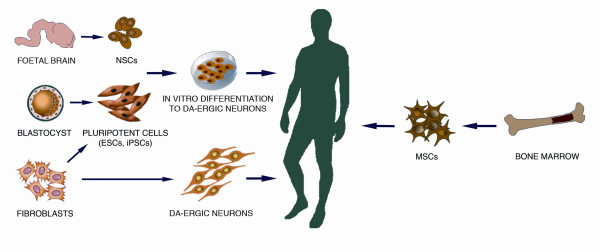
**Schematic illustration of possible sources of stem cells for therapy in Parkinson's disease**. 1) Neural stem cells (NSCs) from human fetal brain, expanded and differentiated to DA-ergic neurons; 2) Pluripotent cells generated from blastocysts (ESCs) or fibroblasts (iPSCs), expanded and differentiated to DA-ergic neurons; 3) DA-ergic neurons generated by direct conversion of fibroblasts; 4) Bone marrow-derived mesenchymal stem cells (MSCs).

Series of studies in PD patients with intrastriatal grafts of human fetal ventral mesencephalic (hfVM) tissue have provided proof-of-principle that cell therapy can work in PD patients, that is, that the dead DA neurons can be replaced by new neurons by transplantation [2-5]. The grafts can provide DA-ergic reinnervation of the striatum and symptomatic relief lasting as long as 16 years following transplantation in some patients [2-5] (Figure 1). The most successful operated cases were able to withdraw from L-DOPA therapy. Although some results were promising, the outcomes across the different clinical trials using hfVM tissue have been inconsistent. In addition, the further development of this approach was hindered by the occurrence of adverse effects, so-called graft-induced dyskinesias (GIDs), in a subgroup of patients [6-8]. Even if new, optimized protocols would improve the safety and efficacy in future hfVM tissue trials, it is doubtful whether transplantation of hfVM tissue will become a mainstream treatment for PD due to shortcomings with tissue availability and standardization of the grafts. In this respect, stem cells (SCs) could provide an unlimited source of well-characterized DA neurons for transplantation and therefore overcome these issues (Figure 2). Here, we aim to discuss what we learned from clinical research with hfVM tissue and also review the current status of SC therapy in PD.

### Lessons learned from hfVM tissue clinical trials

Short- and long-term follow-up studies on PD patients subjected to hfVM tissue transplantation have indicated ways of improving the safety and efficacy in future human cell therapy trials. The correct selection of PD patients entering these trials will be of major importance. Positron emission tomography (PET) studies have indicated that the PD patients with the best functional outcome after intrastriatal transplantation of hfVM tissue were the ones in whom the DA-ergic denervation preoperatively was restricted to the dorsal parts of the striatum [9,10]. Based on these findings, PD patients with more widespread preoperative DA-ergic denervation, including the ventral striatum, should probably be excluded from these trials, as the predictive outcome would be modest or no clinical benefit.

An important breakthrough was the unveiling of one important mechanism underlying GIDs. Studies utilizing PET and clinical observations in previously transplanted PD patients provided strong evidence that GIDs were caused by a graft-derived striatal serotonergic hyperinnervation, possibly engaging in false DA release, which was also weighted by an unfavorable serotonin/DA transporter ratio [4,5,11]. Interestingly, the occurrence of GIDs shows a slow and gradual increase compared to the rapid improvement of motor symptoms following transplantation. This difference is likely due to interactions of serotonin and DA neuronal transmissions because of the gradual expansion of graft-derived serotonergic innervation that takes a leading role in regulating synaptic DA levels in the reinnervated areas [5,12,13].

These findings suggested strategies for avoiding the development of GIDs following DA cell therapy with hfVM tissue or SCs in PD. HfVM tissue contains both dopaminergic and serotonergic neuroblasts [14] and the dissection of hfVM tissue should, therefore, be performed in a way to minimize the serotonergic component. Also, when producing DA neurons from SCs, serotonergic neurons should be kept to a minimum or removed by cell sorting. Moreover, culture and storage of the tissue prior to transplantation could change its composition in favor of non-DA-ergic cells [15]. In accordance, previous studies have reported an increased prevalence of GIDs in patients who received tissue that had been stored for long periods compared to those who received fresh tissue [6,7]. Alternatively, GIDs could be effectively suppressed with systemic administration of serotonin 1A agonists, which dampen transmitter release from serotonergic neurons [4,5,11].

### Stem cell therapy in Parkinson's disease

It remains to be shown whether SC-derived DA neurons can efficiently reinnervate the striatum and provide functional recovery in PD patients. Knowledge from hfVM tissue transplantation in animals and humans has provided a number of requirements for establishing a clinically competitive SC-based therapy in PD. The SC grafts should (a) exhibit a regulated release of DA and molecular, electrophysiological, and morphological properties similar to those of substantia nigra neurons [16,17]; (b) enable survival of more than 100,000 DA neurons per human putamen [18]; (c) reestablish the DA network within the striatum and restore the functional connectivity with host extra-striatal neural circuitries [19]; (d) reverse the motor deficits resembling human symptoms in animal models of PD and induce long-lasting and major symptomatic relief in PD patients; and (e) produce no adverse-effects such as tumor formation, immune reactions and GIDs.

To date, only a few steps have been established towards these goals *in vivo*. The *in vitro *generation of SC-derived cells having DA-ergic properties from fetal brain and embryonic SCs (ESCs) and from bone marrow SCs has already been shown [20-22] (Figure 2). However, it is unclear whether these cells having DA-ergic properties can be used in PD patients. Table 1 summarizes the advantages and disadvantages of different stem cell types for use in PD.

**Table 1:** Advantages and disadvantages of different stem cell types for use in Parkinson's disease

Stem Cell Type	Definition	Advantages	Disadvantages
Embryonic Stem Cells (ESCs)	Pluripotent stem cells derived from the inner cell mass of the blastocyst that are able to differentiate into cells of the three germ layers and show an extensive capability for self-renewal without differentiation, both *in vivo *and *in vitro*	(a) Highly proliferative/retain pluripotency after *in vitro *expansion (b) Can generate DA-ergic neurons (c) Shown to survive transplantation and generate some degree of functional recovery	(a) Risk of tumor formation
Induced pluripotent Stem Cells (iPSCs)	Reprogrammed adult murine fibroblasts into ESC-like cells	(a) Generation of unlimited PD patient-specific cells/autologous transplantation (b) Shown to survive transplantation and generate some degree of functional recovery (c) Could minimize immune reactions and ethical issues	Risk of tumor formation (b) Autologous transplantation - risk of susceptibility to the original pathology of the patient
Bone marrow-derived stromal cells and mesenchymal Stem Cells (MSCs)	Small population of cells in the bone marrow that can differentiate into adipocytes, chondrocytes and osteoblasts, both *in vivo *and *in vitro*	(a) Improve motor performance in mice (b) No adverse effects in humans at 12 months following transplantation	(a) Modest clinical improvement in humans
Fetal brain neural Stem Cells (NSCs)	Multipotent stem cells that are able to differentiate into neurons, astrocytes and oligodendrocytes	(a) Lower risk of tumor formation and immune rejection than ESCs	(a) Shown only limited differentiation *in vivo* (b) Shown only partial effect in PD-like symptoms

ESCs are highly proliferative and retain pluripotency after extended periods of *in vitro *expansion [23]. Since they can give rise to any type of cell in the body including DA-ergic neurons [24-26], their potential to be useful in a clinical setting seems to be great. Rodent and human ESC-derived DA-ergic neurons have been shown to survive transplantation into the striatum of PD rats and generate some degree of functional recovery [27-30]. However, studies have shown that the survival of ESC-derived DA-ergic neurons post-transplantation is relatively low [27,28,31]. A major concern with using ESC-derived DA-ergic neurons for transplantation in PD patients is the risk of adverse effects such as tumor formation which has been reported in rats [29,31]. Cell sorting or prolonged differentiation and thereby exhaustion of non-differentiated cell pools *in vitro *prior to transplantation could potentially reduce the risk of tumor formation [32].

Another promising source of SCs is adult fibroblasts that are reprogrammed to so-called induced pluripotent SCs (iPSCs) [33] and then differentiated to DA-ergic neurons (Figure 2). The iPSC technology has raised the possibility of generating an unlimited source of PD patient-specific DA-ergic neurons, which theoretically also could be used for autologous transplantation [34-37]. DA-ergic neurons were first generated from mouse iPSCs, transplanted into the striatum of a rat PD model and shown to ameliorate functional deficits [36]. Recently, DA-ergic neurons were also produced from iPSCs derived from fibroblasts in adult humans [38,39] and PD patients [37,40]. Such neurons survived transplantation into the striatum of PD rodents and produced some degree of functional recovery [39,40]. Potential advantages with the use of iPSCs are that PD patient-specific DA neuroblasts could minimize the immune reactions and eliminate the ethical issues associated with the use of human ESCs. However, as with ESCs, the risk for tumor formation needs to be minimized before iPSC-derived DA-ergic neurons can be considered as an option for transplantation in a clinical setting in PD. Moreover, there are concerns about whether the DA-ergic neurons delivered by autologous transplantation in PD would be more susceptible to the disease pathology because genetic mutations could also be present in the fibroblast-derived cells [41,42].

Functional DA-ergic neurons with a substantia nigra phenotype can now also be generated by directly reprogramming mouse and human fibroblasts by expressing neural lineage-specific transcription factors [43-45] (Figure 2). This conversion does not occur through a pluripotent SC stage and thereby the risk of tumor formation is eliminated. Before their clinical use in PD is considered, it is necessary to show that the directly converted DA-ergic neurons can survive transplantation and give rise to substantial improvements in animal models.

Fetal brain neural SC (NSC)-derived DA-ergic neurons (Figure 2) are associated with lower risk of tumor formation and immune rejection than ESCs [46]. Early studies reported that non-differentiated NSCs taken from a human source and transplanted in rats have limited differentiation *in vivo *and only partially affect PD-like symptoms [47]. A more recent study showed that non-differentiated NSCs implanted into PD primates survived, migrated, and had a functional impact [48]. A small number of NSC progeny differentiated into DA phenotypes. The use of developmental signals such as sonic hedgehog, Wnt5a and others in fetal NSC differentiation *in vitro *enhances the DA-ergic yield and multiple signals can have synergistic effects [49-54]. Production of fetal NSC-derived DA-ergic neurons through well-controlled differentiation protocols *in vitro *should ensure better homogeneity between grafts.

Bone marrow-derived stromal cells and mesenchymal SCs (MSCs) have been proposed as potential cell sources for transplantation in PD (Figure 2). It has been reported that non-differentiated murine MSCs are able to differentiate into tyrosine hydroxylase-positive neurons and improve motor performance in mice [55]. Also, it has been demonstrated that cells with DA-ergic properties can be produced from both rat and human MSCs, and that transplantation of these cells gave rise to improvement of motor function in an animal model of PD [20]. More recently, a clinical trial in advanced PD patients using unilateral transplantation of autologous bone marrow-derived MSCs into the sublateral ventricular zone reported modest clinical improvement with no adverse effects such as tumor formation at 12 months [56]. In this trial, there were no PET assessments before and after transplantation in order to determine graft survival or changes of DA-ergic striatal function [57]. Thus, the mechanisms underlying the reported modest improvements are completely unknown. Further preclinical work is needed for investigating the ability of MSCs to differentiate into DA-ergic neurons and to reverse functional deficits in animal models.

## Conclusions

Although the ability to restore function in PD patients by DA-ergic neuron replacement has been demonstrated to some extent with hfVM tissue, the focus is now on producing standardized DA-ergic neuroblasts from SCs for transplantation. ESCs and iPSCs seem the simplest to manipulate towards a DA-ergic fate and to produce large numbers of DA-ergic neurons *in vitro*, but fetal brain NSCs could also be useful for clinical application. Both iPSC-derived and directly converted DA-ergic neurons have one more advantage as they potentially can be used for autologous transplantation in PD patients.

Several important *in vivo *properties, which will be decisive for the success or failure of a clinical trial in PD, remain to be demonstrated for human SC-derived DA-ergic neurons in animal models. These include the ability of the SC-derived DA-ergic neurons to substantially reinnervate striatum, restore DA release and markedly improve PD symptoms. Before going ahead with human trials using transplantation of SC-derived DA-ergic neurons, the risks for tumor formation, immune reactions, and development of GIDs need to be tested and proven minimal. Major research efforts will be needed for the development of a clinically competitive SC-based therapy, which for the first time opens up the possibility for an effective restorative treatment for PD patients.

## List of abbreviations

DA: dopamine; DA-ergic: dopaminergic; ESCs: embryonic stem cells; GIDs: graft-induced dyskinesias; hfVM: human fetal ventral mesencephalic; iPSCs: induced pluripotent stem cells; L-DOPA: L-3,4-dihydroxyphenylalanine; MSCs: mesenchymal stem cells; NSCs: neural stem cells; PD: Parkinson's disease; PET: positron emission tomography; SCs: stem cells.

## Competing interests

The authors declare that they have no competing interests.

## Authors' contributions

MP and OL are equally responsible for the content of this article. Both authors read and approved the final manuscript.

## Pre-publication history

The pre-publication history for this paper can be accessed here:

http://www.biomedcentral.com/1741-7015/10/1/prepub
